# Options in human papillomavirus (HPV) detection for cervical cancer screening: comparison between full genotyping and a rapid qualitative HPV-DNA assay in Ghana

**DOI:** 10.1186/s40661-017-0041-1

**Published:** 2017-03-03

**Authors:** Dorcas Obiri-Yeboah, Yaw Adu-Sarkodie, Florencia Djigma, Kafui Akakpo, Ebenezer Aniakwa-Bonsu, Daniel Amoako-Sakyi, Simpore Jacques, Philippe Mayaud

**Affiliations:** 1 Department of Microbiology and Immunology, School of Medical Sciences, University of Cape Coast, Cape Coast, Ghana; 2 Department of Clinical Microbiology, School of Medical Sciences, Kwame Nkrumah University of Science and Technology, Kumasi, Ghana; 3 Laboratory of Molecular Biology and Genetics (LABIOGENE), University of Ouagadougou, Ouagadougou, Burkina Faso; 4 Department of Pathology, School of Medical Sciences, University of Cape Coast, Cape Coast, Ghana; 5 Department of Clinical Research, Faculty of Infectious and Tropical Diseases, London School of Hygiene and Tropical Medicine, London, UK

**Keywords:** Human papillomavirus (HPV), *care*HPV, Genotyping, Cervical cancer, Screening, HIV, Ghana

## Abstract

**Background:**

Modern cervical cancer screening increasingly relies on the use of molecular techniques detecting high-risk oncogenic human papillomavirus (hr-HPV). A major challenge for developing countries like Ghana has been the unavailability and costs of HPV DNA-based testing. This study compares the performance of *care*HPV, a semi-rapid and affordable qualitative detection assay for 14 hr-HPV genotypes, with HPV genotyping, for the detection of cytological cervical squamous intraepithelial lesions (SIL).

**Methods:**

A study comparing between frequency matched HIV-1 seropositive and HIV-seronegative women was conducted in the Cape Coast Teaching Hospital, Ghana. A systematic sampling method was used to select women attending clinics in the hospital. Cervical samples were tested for HPV by *care*HPV and Anyplex-II HPV28 genotyping assay, and by conventional cytology.

**Results:**

A total of 175 paired results (94 from HIV-1 seropositive and 81 from HIV-seronegative women) were analyzed based on the ability of both tests to detect the 14 hr-HPV types included in the *care*HPV assay. The inter-assay concordance was 94.3% (95%CI: 89.7–97.2%, kappa = 0.88), similar by HIV serostatus. The *care*HPV assay was equally sensitive among HIV-1 seropositive and seronegative women (97.3% vs. 95.7%, *p* = 0.50) and slightly more specific among HIV-seronegative women (85.0% vs. 93.1%, *p* = 0.10). *care*HPV had good sensitivity (87.5%) but low specificity (52.1%) for the detection of low SIL or greater lesions, but its performance was superior to genotyping (87.5 and 38.8%, respectively). Reproducibility of *care*HPV, tested on 97 samples by the same individual was 82.5% (95%CI: 73.4–89.4%).

**Conclusions:**

The performance characteristics of *care*HPV compared to genotyping suggest that this simpler and cheaper HPV detection assay could offer a suitable alternative for HPV screening in Ghana.

## Background

Persistent infection with high-risk oncogenic human papillomavirus (hr-HPV) genotypes is aetiologically linked with cervical cancer and its precursor histological cervical intraepithelial neoplasia (CIN) or cytological squamous intraepithelial lesions (SIL) [1, 2]. Modern cervical cancer screening increasingly relies on the use of HPV testing in developed countries because of its high sensitivity to detect CIN/SIL [3, 4]. Resource intensive molecular methods, such as genotyping using polymerase chain reaction (PCR) are able to detect and type HPV but are mostly unavailable in developing countries like Ghana. However, simplified molecular assays are becoming available which will enable HPV molecular diagnosis in resource-constrained settings. The *care*HPV assay (Qiagen, Gaithersburg, MD), a simplified version of the better known Digene Hybrid Capture 2 (HC2), has shown promise with high sensitivity and specificity against histological end points when tested in diverse setting and heterogeneou populations as Africa and China [5–7]. A study conducted among 149 women living with HIV in Burkina Faso and South Africa was the first head to head evaluation of *care*HPV versus HC2 among African women and reported an excellent agreement between the two tests (94.6%, 95% confidence interval [CI]: 89.7 to 97.7%, Kappa value = 0.88) and concluded that *care*HPV assay could be as suitable as HC2 for cervical cancer screening among HIV-infected African women [6].

In Ghana, HPV testing has remained confined to research laboratories where genotyping is used. One such genotyping assay is the recently developed Anyplex^TM^ II HPV28 (Seegene, Seoul, Korea). The assay detects 28 HPV genotypes including 19 hr-HPV types of which 13 are considered carcinogenic (HPV16, 18, 31, 33, 35, 39, 45, 51, 52, 56, 58, 59 and 68), six possible carcinogenic (HPV26, 53, 66, 69, 73 and 82), and nine low-risk HPV types (HPV6, 11, 40, 42, 43, 44, 54, 61 and 70), according to the Interagency for Research on Cancer (IARC) classification [8]. The addition of a high performing, semi-rapid and affordable test such as *care*HPV would considerably enhance access to HPV-based cervical cancer screening in the country. In particular, since HPV molecular assays have very high negative predictive value for the detection of cervical cancer lesions, they could become very useful as a primary screening test or as triage in combination with cytology. However, because they cannot distinguish between transient and persistent infections, their specificity is low. They are thus recommended for use among women aged 30 years and older when most HPV infections should have cleared. Molecular tests can also indicate complete viral eradication if the result is negative 12 months following cervical lesions treatment, hence they can be useful for patients’ follow-up. Studies have compared various strategies including those which combine HPV genotyping with concurrent cytology and those which offer HPV screening without concurrent cytology. The results of the ATHENA trial conducted among 42,209 women in the United States of America comparing various single or combination screening strategies suggest that both strategies are feasible and have equivalent performance depending on factors like age of the woman [9]. Other important factors in selecting a particular screening strategy include its ability to restrict the number of unnecessary colposcopies while maintaining a high negative predictive value [7, 10].

The present study aimed at comparing *care*HPV with HPV genotyping for the molecular diagnosis of HPV and at evaluating the performance of both assays against cytology among HIV-1 seropositive and HIV-seronegative women in Ghana. This is an essential step as the country is looking to inform the development of its cervical cancer screening algorithms.

## Methods

### Study design and subjects

Participants were recruited as part of a larger HPV/cervical cancer epidemiological study (comparing HIV-1 seropositive to HIV-seronegative women) conducted in the Cape Coast Teaching Hospital (CCTH) in Ghana. Briefly, a comparative frequency-matched study was conducted in a systematic (1 in 5) sample of women attending the HIV and the general outpatient clinics at CCTH. Every fifth woman aged ≥18 years was systematically selected from the list of attendants, starting by a randomly selected attendance number for the first woman. If a woman was deemed not eligible (i.e. who had previous total abdominal hysterectomy, was menstruating that day, or was pregnant), the next available patient was offered her place, and every fifth woman whence, to a maximum of 10 women per clinic day. Participants who met the inclusion criteria (i.e. aged ≥18 years and willing to be tested for HIV) were given an explanation of the protocol after which written informed consent was obtained. This method was used to recruit the women for the parent study and then a sub set target of about 50% of participants (every other recruited woman) were asked to be part of the *care*HPV evaluation study.

### Clinical sample collection

Gynaecological examination with speculum was performed, during which cervical swabs were collected from the ecto and endocervix targeting the squamo-columnar junction using a DNA PAP^TM^ Cervical Sampler^TM^ and transported in Swab Specimen Collection Kit (Qiagen, Gaithersburg, MD) for genotyping by Anyplex II HPV 28. For *care*HPV testing, the *care*HPV specific brush and transport medium were used (Qiagen, Gaithersburg, MD). Cervical smears were taken for cytology with a cervical brush and immediately alcohol-fixed at the clinic.

### HPV DNA detection

The Anyplex II HPV 28 test was performed from its specific transport medium as per manufacturer’s protocol previously described [11]. The isolation of nucleic acid was by QIAamp DNA Mini kit (Qiagen, USA) as per established protocol by the manufacturer using 500 μl of the sample. The process from DNA extraction to the RT-PCR for a full panel of 96 plate takes at least 6 h to complete. The *care*HPV test was performed on the samples collected into the *care*HPV transport medium. This test is a semi-rapid test designed based on simplification of the Digene HC2 test technology to be used for the detection of the DNA for 14 hr-HPV types (HPV16, 18, 31, 33, 35, 39, 45, 52, 56, 58, 59, 66 and 68; HPV66 being the addition). This test takes 2.5 to 3 h to perform for a 96 well and involves 6 easy-to-follow steps of denaturation, hybridization and capturing, conjugation, washing, additions of substrate and detection with the illuminometer. The results obtained are qualitative for hr-HPV without indicating the specific genotype [7, 12, 13]. In order to verify testing reproducibility, a random 50% of samples were retested without knowledge of prior result.

### Cervical cytology

Cervical smears were prepared in the laboratory following a standardized protocol for Papanicolaou (Pap) staining. Slides were read by a consultant cytopathologist at CCTH using the Bethesda 2001 guidelines for SIL classification [14].

### Statistical analysis

Analysis of *care*HPV performance compared with genotyping was done for 14 hr-HPV genotypes detectable by both tests (HPV16, 18, 31, 33, 35, 39, 45, 51, 52, 56, 58, 59, 66 and 68). Sensitivity, specificity, positive and negative predictive values (PPV and NPV), and Cohen’s Kappa values for agreement between the two tests were calculated with their 95%CI. These were calculated for the total results and then also done separately for HIV-1 seropositive and HIV-seronegative women separately. Data analyses were performed using Stata version 13 software (Stata Corp, Texas USA).

### Ethics

Ethical approval for this study was obtained from the Committee on Human Research Publications and Ethics (CHRPE) of the School of Medical Sciences (SMS), Kwame Nkrumah University of Science and Technology (KNUST) before the study commenced. Study participants were recruited only after obtaining signed written informed consent.

## Results

Overall, 333 eligible women were included in the parent study, 163 HIV-1 seropositive women (mean age 43.8 years, standard deviation [SD] ±9.4) and 170 HIV-seronegative women (mean age 44.3 years, SD ±12.8). A total of 197 paired *care*HPV and genotyping samples from the subsample of women (100 HIV-1 seropositive (mean age 44.7 years, SD ±9.7) and 97 HIV-seronegative (mean age 43.7 years, SD ±12.8) randomly selected into the *care*HPV validation study were tested, and 175 results (89%) were available for analysis based on the ability of both tests to detect the 14 hr-HPV types. For 21 *care*HPV results (6 from HIV-1 seropositive and 15 from HIV-seronegative women), Anyplex II HPV 28 detected genotypes which are undetectable by *care*HPV (i.e., low-risk types, as well as HPV26, 53, 69, 73 and 82). In addition, one *care*HPV sample (from an HIV-seronegative woman) gave an invalid result despite repeat testing. These 22 samples (11.1%) were not included in the analysis of *care*HPV performance.

The hr-HPV prevalence by *care*HPV was 55% (95%CI: 48.0–62.9) overall, 79% (95%CI: 69.0–86.5) among HIV-1 seropositive women and 28.0% (95%CI: 19.0–39.5) among HIV-seronegative women (p ≤ 0.0001) (Table 1). Similarly, the hr-HPV prevalence by genotyping was 57% (95%CI: 49.0–64.0), 79% (95%CI: 69.0–86.5) among HIV-1 seropositive women and 31% (95%CI: 12.0–42.1) among HIV-seronegative women (p ≤ 0.0001).

**Table 1:** Performance characteristics of c*are*HPV assay for the detection of 14 high-risk (hr) HPV genotypes compared with HPV genotyping among 175 women in Cape Coast, Ghana

	All women (*N* = 175) % (95% CI)	HIV-1 seropositive women (*N* = 94) % (95% CI)	HIV seronegative women (*N* = 81) % (95% CI)	^*^ *P*-value
hr-HPV prevalence	55.0 (48.0–62.9)	79.0 (69.0–86.5)	28.0 (19.0–39.5)	0.0001
Sensitivity	96.9 (91.2–99.4)	97.3 (90.6–99.7)	95.7 (78.1–99.9)	0.50
Specificity	91.0 (82.4–96.3)	85.0 (62.1–96.8)	93.1 (83.3–98.1)	0.10
PPV	93.1 (86.2–97.2)	96.0 (88.8–99.2)	84.6 (65.1–95.6)	0.01
NPV	95.9 (88.8–99.2)	89.5 (66.9–98.7)	98.2 (90.3–100.0)	0.02
Agreement	94.3 (89.7–97.2)	94.7 (88.0–98.3)	93.8 (86.2–98.0)	0.77
Kappa value (95%CI)	0.88 (0.81–0.95)	0.84 (0.70–0.98)	0.85 (0.73–0.98)	0.86
*P*-value for Kappa	<0.0001	<0.0001	<0.0001	

*PPV* positive predictive valuem, *NPV* negative predictive value

^*^ comparing HIV-1 seropositive and HIV-seronegative women

There was excellent agreement (94.3%, 95%CI: 89.7–97.2%) between *care*HPV and genotyping overall (kappa = 0.88, 95%CI: 0.81–0.95, *p* < 0.0001), and the agreement was similar among HIV-1 seropositive (94.7%, 95%CI: 88.0–98.3%) and seronegative (93.8%, 95%CI: 86.2–98.0%) women (Kappa of 0.84 and 0.85, respectively) (Table 1). The *care*HPV assay was equally sensitive among HIV-1 seropositive and HIV-seronegative women (97.3% vs. 95.7%, *p* = 0.50) and slightly more specific among HIV-seronegative women (85.0% vs. 93.1%, *p* = 0.10), but these differences were not statistically significant (Table 1).

For 9 of the hr-HPV types (HPV18, 31, 33, 35, 39, 45, 51, 52 and 68), the concordance between *care*HPV and Anyplex II HPV 28 was 100%. For 4 genotypes *care*HPV missed one positive sample, and for HPV58 it missed 2 samples (Table 2).

**Table 2:** Agreement between results of *care*HPV and genotyping with Anyplex II HPV28 for the detection of 14 high-risk HPV genotypes, among 175 women in Cape Coast, Ghana

HPV genotypes	Anyplex II HPV28 No. Positive (%)	*care*HPV No. Positive (%)	Agreement %
16	15 (8.6)	14 (8.0)	93.3
18	15 (8.6)	15 (8.6)	100.0
31	13 (7.4)	13 (7.4)	100.0
33	12 (6.9)	12 (6.9)	100.0
35	17 (9.7)	17 (9.7)	100.0
39	8 (4.6)	8 (4.6)	100.0
45	8 (4.6)	8 (4.6)	100.0
51	1 (0.6)	1 (0.6)	100.0
52	16 (9.1)	16 (9.1)	100.0
56	12 (6.9)	11 (6.3)	91.7
58	20 (11.4)	18 (10.3)	90.0
59	6 (3.4)	5 (2.9)	83.3
66	7 (4.0)	6 (3.4)	85.7
68	12 (6.9)	12 (6.9)	100.0

**Fig. 1:**
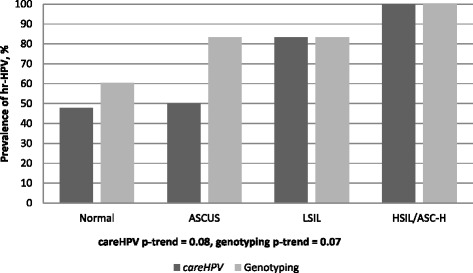
14 hr-HPV prevalence by *care*HPV and genotyping according to cytological findings among women, Cape Coast, Ghana. Cytology readings: Normal = no abnormal findings found; ASCUS = atypical squamous cells of undetermined significance; LSIL = low grade squamous intraepithelial lesions; HSIL = high grade squamous intraepithelial lesions; ASC-H = Atypical squamous cells cannot rule out HSIL. Hr-HPV = high risk HPV types

*care*HPV prevalence increased according to severity of cytological lesions, from 47.8% among women with normal cytology to 100% among women with high-grade lesions (HSIL/ASC-H) (p-trend = 0.08). Similarly, hr-HPV prevalence by genotyping increased by cytological grade severity (p-trend = 0.07) (Fig. 1). *care*HPV and genotyping had the same sensitivity for detection of lesions low SIL and above of 87.5% (95%CI: 43.3–99.7%) but *care*HPV had statistically significantly higher specificity (52.1% vs. 38.8%, 95%CI: 31.8–46.2%, *p* < 0.0001), PPV and NPV than genotyping (Table 3).

**Table 3:** Performance of *care*HPV and genotyping to detect cases of cytological abnormalities (LSIL and greater, *n* = 8) among 197 study participants in Cape Coast, Ghana

Performance Indicators	*care*HPV % (95%CI)	Anyplex II HPV 28 % (95%CI)
Number of LSIL+ cases detected by assay	7	7
Sensitivity	87.5 (47.3–99.7)	87.5 (47.3–99.7)
Specificity	52.1 (44.7–59.5)	38.8 (31.8–46.2)
Positive predictive value (PPV)	7.2 (3.0–14.3)	5.7 (2.3–11.5)
Negative predictive value (NPV)	99.0 (94.5–100.0)	98.6 (92.7–100.0)

A random sample of approximately 50% of all *care*HPV samples was tested twice by the same individual with *care*HPV to check the reproducibility of results. Of these, 80/97 produced the same results giving a reproducibility rate of 82.5% (95%CI: 73.4–89.4%). In 5/97 initially negative *care*HPV results the second result was positive, 2/97 became invalid and in 8/97 positive *care*HPV result, the second result was negative, and 2 became invalid.

## Discussion

Given the high cost and resource intensive nature of genotyping for HPV screening, both in terms of skills and materials, it is essential that developing countries find acceptable alternatives to move into the modern cervical cancer screening era. The advantage of full genotyping is its higher analytical sensitivity and ability to specifically identify the genotypes present in a population. While this is essential for research or epidemiological monitoring purposes, it is not absolutely necessary for clinical care. The role of HPV screening for clinical practice is to help establish a protocol of screening which is cost effective and helps identify women having hr-HPV infection so they can have further evaluation [7], whilst helping reduce the number of unnecessary colposcopies and histology. Since molecular testing of HPV does not necessarily require representative samples from the cervix to be taken, an additional potential benefit is the possible use of self-collected vaginal samples. This might increase testing by women especially in settings where self-collection might be preferred either due to cultural reasons or the convenience of not necessarily having to visit a health facility to provide samples [15–17]. Full genotyping requires DNA extraction (an additional cost) and molecular testing. Samples for DNA extraction for PCR have strict temperature control: they must be immediately extracted or kept in a fridge, and once extracted the DNA must be stored at 20 °C until used. Both DNA extraction and PCR testing require extensive technical training and appropriate setup. There is also the need to ensure continuous supply of electrical power throughout the processing until results are generated. All of these factors pose a tremendous challenge for resource-constrained countries like Ghana.

*care*HPV represents an alternative HPV screening assay that has been specifically developed for resource-constrained settings. This assay requires just bench top and 3 portable equipment which has a backup battery to store power enough to run a full set of 96 samples without the need for an external supply of electricity. The samples for *care*HPV can also be stored at room temperature for up to 4 weeks, do not require DNA extraction and require very limited technical knowledge to be performed. While *care*HPV has been evaluated in some settings, this research presents the first such evaluation done among women including HIV seropositive and seronegative women in Ghana. The simplicity of the assay and its relative robustness in the context of a resource-constrained laboratory setting was confirmed in this study. Various studies have been conducted using this assay including in Uganda [12], rural China [18], rural Thailand [19] with good outcomes.

This study found an excellent agreement (94.3%, *k* = 0.88) between *care*HPV and full HPV genotyping for the detection of 14 hr-HPV genotypes, and the result was similar among HIV-1 seropositive and HIV-seronegative women. The *care*HPV assay was slightly more sensitive among HIV-1 seropositive women but more specific among HIV-seronegative women. Investigators in Burkina Faso and South Africa found very similar excellent agreement (94.6%) for *care*HPV compared to HC2 [6], a well-validated HPV qualitative assay used in many settings, and when compared to genotyping using the InnoLiPA assay [20]. The clinical performance though not extensively investigated in this study was good as *care*HPV detected 83.3% of all cases with LSIL and all cases (100%) of HSIL/ASC-H. Other studies have demonstrated good clinical performance of *care*HPV in HIV-seronegative African women [21] and in African women living with HIV-1. Segondy et al. [20] found the sensitivity and specificity of *care*HPV for the diagnosis of HSIL among 929 HIV-1 seropositive women in Burkina Faso and South Africa to be 88.8 and 61.8% respectively. The negative predictive value of *care*HPV for detection of cytological lesions was 99.0% in this study. This is very important because it implies that it could serve as an essential screening tool. Given its good sensitivity but low specificity, *care*HPV testing might be best performed as triage test with cytology or visual inspection (VIA) to reduce unnecessary referrals for colposcopy. It will be useful to study the cost effectiveness of such strategies in the Ghanaian socio-economic context.

To check reproducibility and hence reliability of results, 97 samples were tested in duplicate in this study by the same individual. Reproducibility was found to be 82.5%. This is good but still implies that all of the 6 processing steps be completed without fault by a meticulous lab technician to reduce the risk of having variable results, and that quality control should be routinely implemented. The positive and negative controls included for each plate serve to ensure that only valid results are read, and only a small proportion of retests we found invalid (4%). To assess the reliability of results in a study evaluating *care*HPV assay in Nigeria, researchers checked intra-rater (reproducibility of results by the same local technician) and inter-rater (reproducibility of results between 2 different local technicians) agreements. Intra-rater agreement was 98.8% (*k* = 0.97) and 98.9% (*k* = 0.97) for Technicians 1 and 2, respectively, and the inter-rater agreement was 96.3% (*k* = 0.90), suggesting that *care*HPV results were reliable [5], which is very encouraging for countries in the region. However, the higher agreement values found in the Nigerian study suggest that this can also be very dependent on locale/staff.

The present study had some limitations. The number of samples tested by *care*HPV was relatively low, owing to financial constraints and dependence on donated *care*HPV kits; and for the same reasons a more extensive repeat testing could not be organized. Furthermore, a full economic evaluation of cervical cancer screening was beyond the scope of this study. However, based on market prices and personal communication with relevant people, the cost of genotyping (including reagents and technician time) would come to approximately US$ 100.00 per sample, whereas *care*HPV cost could be about US$ 15–20.00 per sample. Other forms of cervical screening include cytology (Pap smears) being offered at a minimum of US$ 15.00 in Ghana (personal communication from facilities offering these tests), whereas visual inspection using VIA could cost as little as US$ 5.00 (up to US$ 15) [22]. As indicated by the study by Quentin et al., the feasibility of increasing uptake to achieve economies of scale in Ghana is essential for choosing a screening method [22]. The fact that HPV-based screening protocols can increase screening intervals [23, 24] and allow for possible patient self-collection [25] are important advantages that could reduce costs and increase access for both health services and Ghanaian women.

## Conclusion

This study has demonstrated that *care*HPV has very good concordance with, and good performance characteristics compared to, HPV genotyping for the detection of cervical squamous intraepithelial lesions, and whilst reproducibility could be improved, the findings support the possibility of setting up HPV screening without the need for resource-intensive genotyping as a suitable alternative for cervical cancer screening in countries like Ghana.

## Abbreviations

ASCUS: Atypical squamous cells of undetermined significance
CCTH: Cape Coast Teaching Hospital
CD4+: Activated T-lymphocytes CD4
CI: Confidence interval
CIN: Cervical intraepithelial neoplasia
HIV: Human Immunodeficiency Virus
HPV: Human Papilloma Virus
hr-HPV: High-Risk Human Papilloma Virus
H-SIL: High grade squamous intraepithelial lesions
KNUST: Kwame Nkrumah University of Science and Technology
LSIL: Low grade squamous intraepithelial lesions
PLHIV: People Living with HIV
SIL: Squamous intraepithelial lesions
SSA: Sub Saharan Africa
VIA: Visual Inspection with Acetic acid
