# Erratum to: Options in human papillomavirus (HPV) detection for cervical cancer screening: comparison between full genotyping and a rapid qualitative HPV-DNA assay in Ghana

**DOI:** 10.1186/s40661-017-0044-y

**Published:** 2017-03-23

**Authors:** Dorcas Obiri-Yeboah, Yaw Adu-Sarkodie, Florencia Djigma, Kafui Akakpo, Ebenezer Aniakwa-Bonsu, Daniel Amoako-Sakyi, Jacques Simpore, Philippe Mayaud

**Affiliations:** 1 Department of Microbiology and Immunology, School of Medical Sciences, University of Cape Coast, Cape Coast, Ghana; 2 Department of Clinical Microbiology, School of Medical Sciences, Kwame Nkrumah University of Science and Technology, Kumasi, Ghana; 3 Laboratory of Molecular Biology and Genetics (LABIOGENE), University of Ouagadougou, Ouagadougou, Burkina Faso; 4 Department of Pathology, School of Medical Sciences, University of Cape Coast, Cape Coast, Ghana; 5 Department of Clinical Research, Faculty of Infectious and Tropical Diseases, London School of Hygiene and Tropical Medicine, London, UK

## Erratum

Upon publication of the original article [1], it was noticed that the author’s name “Jacques Simpore” was incorrectly given as “Simpore Jacques”. This has now also been corrected in the original article.
